# Usability and acceptance of a mobile health wallet for pregnancy-related healthcare: A mixed methods study on stakeholders’ perceptions in central Madagascar

**DOI:** 10.1371/journal.pone.0279880

**Published:** 2023-01-03

**Authors:** Etienne Lacroze, Anna Frühauf, Kim Nordmann, Zavaniarivo Rampanjato, Nadine Muller, Jan-Walter De Neve, Ralisimalala Andriamampianina, Elsa Rajemison, Till Bärnighausen, Samuel Knauss, Julius Valentin Emmrich

**Affiliations:** 1 Global Digital Health Lab, Charité Global Health, Charité - Universitätsmedizin Berlin, Berlin, Germany; 2 Heidelberg Institute of Global Health, Medical Faculty and University Hospital, University of Heidelberg, Heidelberg, Germany; 3 Department of Infectious Diseases and Pulmonary Medicine, Charité - Universitätsmedizin Berlin, Berlin, Germany; 4 Ministry of Public Health of the Republic of Madagascar, Antananarivo, Madagascar; 5 National Institute of Public and Community Health, Antananarivo, Madagascar; 6 Department of Global Health and Population, Harvard T.H. Chan School of Public Health, Boston, Massachusetts, United States of America; 7 Africa Health Research Institute (AHRI), Mtubatuba, KwaZulu-Natal, South Africa; 8 Department of Neurology, Charité - Universitätsmedizin Berlin, Berlin, Germany; 9 Berlin Institute of Health, Berlin, Germany; WCU: Wachemo University, ETHIOPIA

## Abstract

**Background:**

Several sub-Saharan African countries use digital financial services to improve health financing, especially for maternal and child health. In cooperation with the Malagasy Ministry of Health, the NGO Doctors for Madagascar is implementing a mobile health wallet for maternal health care in public-sector health facilities in Madagascar. Our aim was to explore the enabling and limiting factors related to the usability and acceptance of the Mobile Maternal Health Wallet (MMHW) intervention during its implementation.

**Methods:**

We conducted a cross-sectional, mixed methods study with mothers and pregnant women and facility- (FBHWs) and community-based (CHWs) health workers from public-sector health facilities in three districts of the Analamanga region in Madagascar. We used a convergent design in collecting and analyzing quantitative and qualitative data. We performed one-stage proportional sampling of women who had signed up for the MMHW. All FBHWs and CHWs at primary care facilities in the intervention area were eligible to participate.

**Results and significance:**

314 women, 76 FBHWs, and 52 CHWs were included in the quantitative survey. Qualitative data were extracted from in-depth interviews with 12 women and 12 FBHWs and from six focus group discussions with 39 CHWSs. The MMHW intervention was accepted and used by health workers and women from different socioeconomic backgrounds. Main motivations for women to enroll in the intervention were the opportunity to save money for health (30.6%), electronic vouchers for antenatal ultrasound (30.2%), and bonus payments upon reaching a savings goal (27.9%). Main motivation for health workers was enabling pregnant women to save for health, thus encouraging facility-based deliveries (57.9%). Performance-based payments had low motivational value for health workers. Key facilitators were community sensitization, strong women-health worker relationship, decision making at the household level, and repetitive training on the use of the MMHW. Key barriers included limited phone ownership, low level of digital literacy, disinformation concerning the effects of the intervention, and technical problems like slow payout processes.

## Introduction

### Background

While many sub-Saharan African (SSA) countries have introduced user fee exemption policies for maternal and child health, out-of-pocket payments (OOP) continue to put expectant mothers and their families at risk of catastrophic and impoverishing health expenditure [1, 2]. This is a pertinent issue for mothers and families from low- or middle-income households whose resources are often insufficient to cover the costs of care [3]. Financial barriers are a main contributor to reduced access to care, and can result in increased morbidity and mortality during pregnancy and childbirth [4]. Arguably, this situation has been exacerbated by the COVID-19 pandemic [5–7].

Mobile money services rely on Unstructured Supplementary Service Data (USSD) to store, send and receive money on any mobile phone without the need for a bank account or internet access. Digital financial services using mobile money improve health financing in several SSA countries and the COVID-19 pandemic has further increased the popularity of these technologies [8, 9]. Most mobile money-based interventions support revenue collection and purchasing. Examples include remote insurance enrollment and premium payments in Ghana and Kenya [10, 11]; electronic saving platforms and mobile wallets in Kenya, Madagascar, and Zimbabwe [12–14]; and payments, credits, and loans for health in many countries. Compared with non-users, mobile money users have an overall lower risk of catastrophic health expenditure during emergency care and are less likely to reduce non-medical expenses for education or food [15]. This may be explained by mobile money users being able to redistribute funds more efficiently, receive more remittances from third parties, and save money more easily on a mobile wallet. Yet the uptake of mobile money for health financing has yielded mixed results, which caused its equity and efficiency to be called into question. Research on the design, implementation, and outcome of these interventions is limited [16–20].

#### Maternal health and digital financial services in Madagascar

In Madagascar, one of the least developed countries, financial factors are a major barrier to accessing skilled maternal health care [21, 22]. Only around 50% of pregnant women complete four antenatal care visits and over half of the deliveries take place without qualified health personnel [21, 23]. The national maternal mortality rate in 2017 was 335 per 100,000 live births but estimated to be up to three times higher in the poorest districts of Madagascar [21]. OOP represent 24.7% of total health care expenditure and the risk of catastrophic health expenditure during pregnancy is high [24, 25].

Opportunities for digital financial services for health are mirrored in Madagascar: In the last decade, mobile phone subscription rates have risen more than 13-fold while the number of mobile money accounts has overtaken that of formal bank accounts [26, 27]. In prior work, we found a high degree of perceived usefulness of mobile money-based savings and payments for maternal health care among key stakeholders and pregnant women in the Malagasy capital Antananarivo, particularly those from low-income households [13].

#### The Mobile Maternal Health Wallet

Against this backdrop, the non-governmental organization Doctors for Madagascar developed a mobile health wallet for maternal health care to mitigate or eliminate OOP for health expenditure. We described the Mobile Maternal Health Wallet (MMHW) in detail elsewhere [13, 28]. Briefly, the MMHW allows expectant mothers to save, pay, and receive mobile money and electronic vouchers for maternal health services. The MMHW can be accessed from any mobile phone via the USSD menu. In cooperation with the Malagasy Ministry of Health, Doctors for Madagascar has implemented the MMHW intervention in 25 public-sector primary care facilities and 2 referral hospitals in Antananarivo, central Madagascar, from which data for this research was drawn.

This mixed-methods study explored the enabling and limiting factors related to the usability and acceptance of the MMHW during the implementation of the pilot intervention. We used a one-stage cluster design to randomly select women who enrolled for the MMHW between July and September 2019 for our quantitative survey. To explain and complete the quantitative results, we conducted in-depth interviews (IDIs) and semi-structured focus group discussions (FGDs) to elicit stakeholders’ perspectives on the MMHW. The pilot intervention started in October 2018 and is currently ongoing as of August 2022. Results from the pilot will inform a larger cluster randomized trial in the study area to determine the impact of the MMHW on health outcomes as well as its cost-effectiveness [28].

## Materials and methods

### Study setting

Madagascar has a population of 27.7 million [29], of which 80.5% [30] live in rural areas. The poverty headcount ratio at USD1.90 a day (2011 purchasing power parity) is 78.8%; gross domestic product per capita is USD 523 [29]. Madagascar ranks 164th out of 189 in the Human Development Index [31].

**Fig 1:**
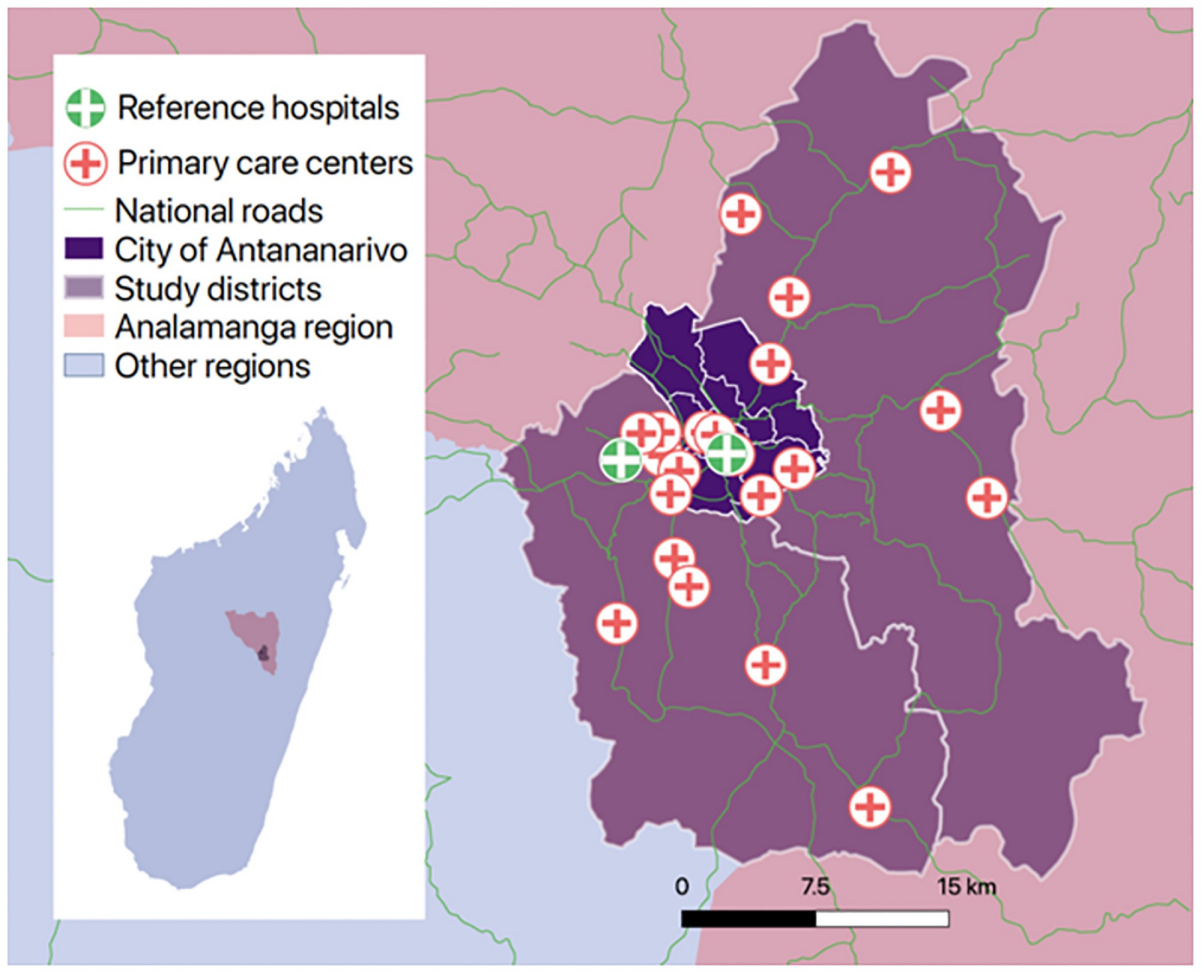
Map depicting the study area of the Mobile Maternal Health Wallet intervention in Analamanga, Madagascar.

The study was conducted in three rural, peri-urban, and urban districts (Atsimondrano, Avaradrano, and Renivohitra) of the Analamanga region of Madagascar including the capital Antananarivo. The study area included 61 public-sector primary care facilities and four public reference clinics. Of those, 22 primary care facilities and two reference clinics were included in the MMHW intervention at the time of study (Fig 1).

### Study design

We conducted a cross-sectional, mixed-methods study employing a convergent design in collecting and analyzing quantitative and qualitative data simultaneously [32]. We used a retrospective triangulation protocol to validate the findings generated by each method through the evidence produced by the other. We collected and analyzed quantitative data to measure the prevalence of multiple characteristics relevant to barriers and facilitators of the intervention among all stakeholders. Qualitative IDIs and FGDs with selected stakeholders were chosen to gain a deeper understanding of the challenges, their implications, and which facilitators need to be promoted during the scale up of the MMHW in a subsequent randomized trial.

### Eligibility criteria

All mothers and pregnant women over 18 years of age or emancipated minors, who enrolled for the MMHW between July 1 and September 30, 2019, were eligible to participate. Women unwilling or unable to consent were excluded.

All facility- (FBHWs) and community-based health workers (CHWs) of legal age (at least 18 years old) at any of the primary care facilities in the MMHW intervention area were eligible to participate. We excluded FBHWs and CHWs who were minors, unable to provide consent, or not involved in the MMHW intervention.

### Implementation of the Mobile Maternal Health Wallet

The core system behind the MMHW is a software that allows users (mothers and pregnant women) to save and pay for health care services using mobile money and to receive electronic vouchers that can be redeemed at participating health facilities.

To incentivize pregnant women to save money within their MMHW, the implementing NGO paid a bonus to women who reached a savings target. After delivery, unspent money was disbursed. Additional incentives included vouchers for ambulance services and obstetric ultrasound at no cost.

FBHWs informed and enrolled pregnant women during routine antenatal care visits. CHWs informed and enrolled pregnant women during household visits and community sensitization activities. CHWs and FBHWs received a performance-based payment upon successful user enrollment. A toll-free hotline on MMHW-relevant topics was available to the public.

### Quantitative methodology

We developed three quantitative surveys to collect information on the experiential characteristics of the MMHW intervention and its implementation from women, FBHWs and CHWs. To characterize the socio-demographic characteristics of women using the MMHW, we elicited information on household income, use of mobile phones and mobile money, and savings behavior. Experiential characteristics were assessed by asking about usability, perception and satisfaction with the MMHW. The questionnaires were prepared in English, professionally translated into Malagasy, and translated back into English to confirm adequate translation.

### Sampling and randomization

**Fig 2:**
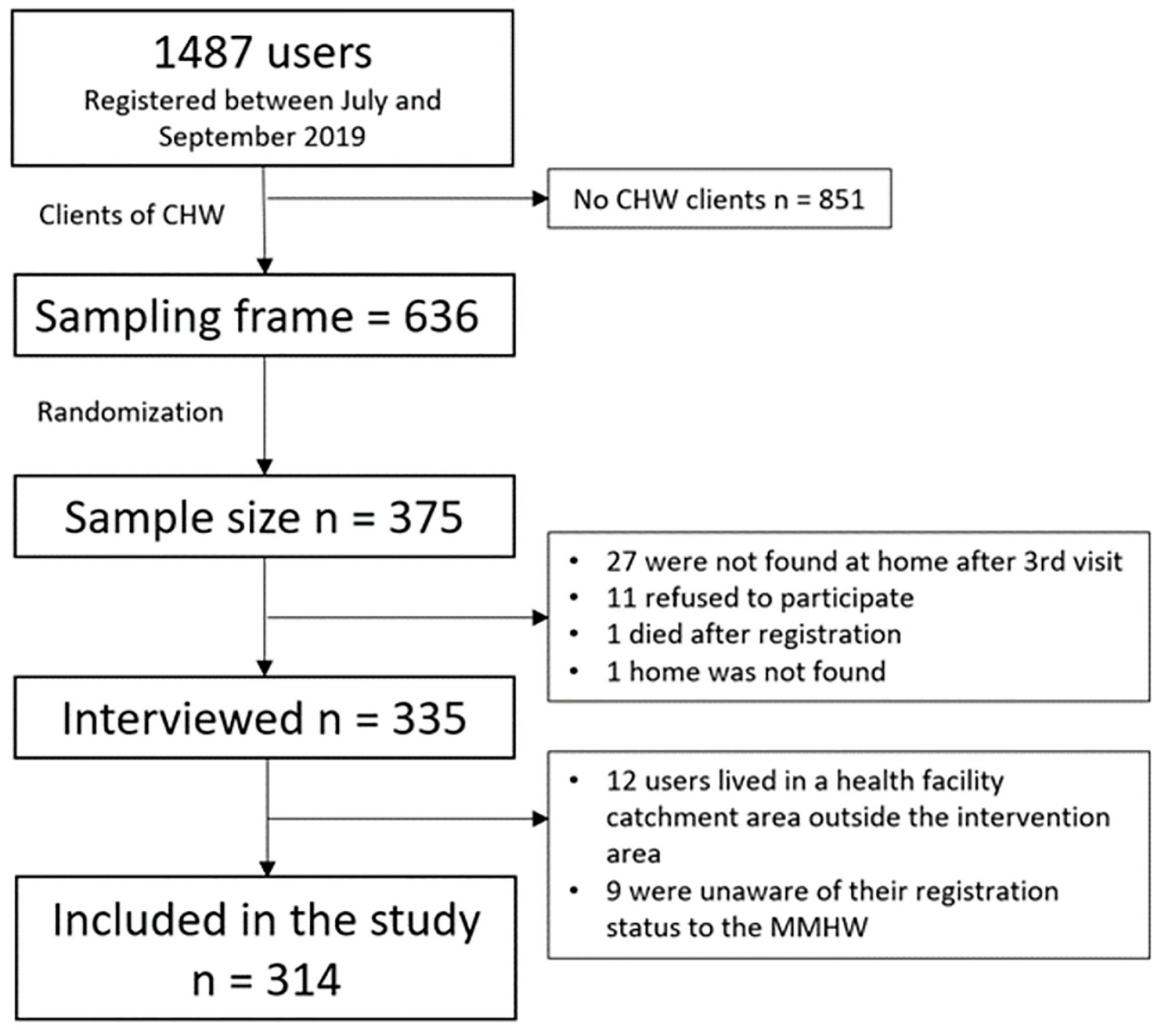
Flowchart of recruitment of women who enrolled for the MMHW intervention between July 1 and September 30, 2019 in Analamanga, Madagascar. CHW, community health worker; MMHW, Maternal Mobile Health Wallet.

We obtained a proportionate random sample of women who enrolled for the MMHW intervention between July 1 and September 30, 2019 (n = 1,487). We excluded all women unknown to any of the CHWs in the intervention area as they could not be reached for data collection (n = 851), yielding a sampling frame of 636 women (Fig 2). Using the web-based calculator OpenEpi Version 3 [33], with a 95% CI, a 0.05 significance level, and an anticipated frequency of 50%, a sample size of 240 was deemed sufficient. As women in Madagascar usually travel to the countryside after giving birth, we assumed that around one third of women may not be found to conduct an interview. Therefore, we included 375 women in our final sample. From each health facility (n = 22) we randomly selected 59% (375/636) of users based on MMHW enrollment data to participate in the quantitative survey. All FBHWs (n = 76) and CHWs (n = 52) who met the eligibility criteria were included and completed the quantitative survey. Blinding was not feasible because all mothers and pregnant women received the intervention.

### Data collection

Data were collected between November 12, 2019, and March 2, 2020. Quantitative surveys were led by bilingual (French, Malagasy), female data collectors, who were experienced in collecting quantitative data and had a university degree in natural sciences. They were trained on i) the background and intentions of the study, ii) use of the digital device (Tablet) to collect data, iii) ethical issues and consent administration prior to field work. Data collectors visited the women at home. If women were not present or did not have time, two follow-up visits were conducted. Data collectors visited FBHWs and CHWs at their assigned health centers. Eligible participants were informed about the study and informed consent was obtained prior to study enrolment. All surveys were conducted in a separate room to ensure confidentiality during the interview and lasted between 10 and 35 minutes. Data were collected digitally by REDCap software between November 12, 2019 and March 2, 2020 [34]. During data collection, a supervisor monitored data quality by cross-checking for duplicate entries, out-of-range values, and overall consistency.

### Data analysis

We analyzed data separately for women, FBHWs, and CHWs using descriptive statistics to summarize key findings. Quantitative data was analyzed using IBM SPSS Statistics, Version 26.0.

### Qualitative methodology

We developed qualitative semi-structured interview guides for each of the three user groups. We conducted IDIs with participating women and organized FGDs with FBHW and CHW. The topic areas of the semi-structured IDIs and FGDs were: 1) motivation and perception, 2) experience with the MMHW, 3) challenges in use, and 4) factors that facilitate or hinder use.

### Sampling

We used criterion-based purposeful sampling based on data from the quantitative survey to obtain diverse samples of women and FBHWs for qualitative interviews. Selection criteria for women included general satisfaction with the MMHW (least satisfied vs. medium satisfied vs. most satisfied), status of pregnancy (pregnant vs. delivered), and household income. We selected FBHWs based on their health facilities level of satisfaction with the MMHW intervention (least satisfied vs. most satisfied). A key strength of this approach was to capture large variability in perceptions and experiences. All staff members of the two least and two most satisfied health centers that met the inclusion criteria were interviewed. All CHWs who participated in the quantitative survey were invited to participate in FGDs of 4–8 participants each.

### Data collection

Qualitative data collection was implemented between December 10, 2019, and January 17, 2020 by a Malagasy medical doctor and a sociologist with several years of experience in conducting qualitative research. Both interviewers were trained on i) the background and intentions of the study, ii) use of the semi-structured interview guides, iii) adaptation of interview styles by category of study respondents, iv) systematic probing, and v) ethical issues and consent administration prior to field work. All qualitative interviews were accompanied by a second researcher who recorded observations and debriefed the interviews with the interviewer. Interviewers visited women at their residence. FBHWs and CHWs were interviewed at the health facility. Eligible participants were informed about the study and informed consent was obtained prior to study enrolment. IDIs and FDGs were recorded digitally and lasted between 15 and 40 minutes and 50 and 70 min, respectively. Study instruments included sections on user-experiences with registration, saving and payment processes as well as saving habits, user-satisfaction, and perceived benefits.

### Data analysis

Malagasy research assistants transcribed the audio recordings verbatim in the original language. Professional translators translated the transcription into English. Data analysis was conducted separately from data collection. Only the observer during the qualitative Interviews (EL) was involved in the data analysis as well. Dedoose software (Dedoose (Dedoose Version 9.0.17, Los Angeles, CA: SocioCultural Research Consultants, LLC) was used for qualitative data analysis. Based on three randomly sampled IDIs, two researchers (EL, KN) jointly developed a codebook for thematic analysis. Then, all IDIs and FGDs were manually coded independently by both researchers using an inductive approach and the same codebook. After six analyzed interviews, both researchers compared their coding, discussed differences, came to a consensus, and adapted or extended the codebook by new codes that emerged from the transcripts. Once all interviews had been double-coded and compared, the individual excerpts were analyzed again by both researchers based on the final codebook and reassigned, if necessary. Emerging themes and subthemes with potential influence on the acceptance or usability of the MMHW intervention were grouped together and are presented accordingly in the results section.

### Research ethics approval

The study was approved by Heidelberg University Hospital Ethics Committee (No. S-212/2018) and the Malagasy Ministry of Health. All participants provided written informed consent before enrollment. All users were free to participate in the study and could withdraw at any time without repercussions.

## Results

In total, 314 women, 76 FBHWs (28 midwives, 22 pharmacists, 22 physicians, and 4 nurses) and 52 CHWs were included in the quantitative survey. Qualitative data were extracted from IDIs with 12 women and 12 FBHWs and six FGDs with 39 CHWs. Women’s socio-demographic characteristics are summarized in Table 1.

**Table 1:** Socio-demographic characteristics and previous experiences with mobile money of mothers and pregnant women participating in this study (N = 314).

	n	% (95% CI)	Mean (±SD)	Median (min-max)
**Age, median in years** (N = 310)				25 (16–45)
**Age group in years** (N = 310)				
16–20	69	22.3		
21–25	94	30.3		
26–30	80	25.8		
31–35	41	13.2		
36–40	24	7.7		
41–45	2	0.6		
>45	0	0		
**Children per woman** (N = 306)			1.8 (±1.3)	
**Marital status** (N = 314)				
Married	268	85,4 (81.4–89.3)		
Partnership	33	10.5 (7.1–13.9)		
Single	4	1.3 (0.0–2.5)		
Divorced	3	1.0 (0.0–2.0)		
**Highest education level completed** (N = 310)				
No formal education	4	1.3 (0.0–2.5)		
Primary school	107	34.5 (29.2–39.8)		
Secondary school	151	50.3 (44.7–56.0)		
Higher education (e.g., University)	48	15.5 (11.5–19.5)		
**Occupation** (N = 307)				
Unemployed	157	51.7 (45.5–56.7)		
Vendor	53	17.3 (13.0–21.5)		
Farmer	27	8.8 (5.6–12.0)		
Auxiliary	11	3.6 (1.5–5.7)		
Other	59	19.2 (14.8–23.6)		
**Number of household members** (N = 307)			4.3 (±1.6)	
**Television at home** (N = 309)				
Yes	183	59.2 (53.7–64.7)		
No	126	40.8 (35.3–46.3)		
**Electricity at home** (N = 310)				
Yes	208	67.1 (61.9–72.3)		
No	102	32.9 (27.7–38.1)		
**Monthly Income in MGA** (N = 251)				
< 50,000	27	10.8 (6.9–14.6)		
50,000–99,999	47	18.7 (13.9–23.6)		
100,000–199,999	59	23.5 (18.3–28.8)		
200,000–399,999	81	32.3 (26.5–38.1)		
> 400,000	37	14.8 (10.4–19.1)		
Household income in MGA (N = 251)			221,287 (±242,041)	160,000 (5,000–2,100,000)
Household get regular income during last six months (N = 300)	184	61.3 (55.8–66.8)		
**Phone access** (N = 300)				
Own phone	150	50.0 (44.3–55.7)		
Phone in family	127	40.6 (35.1–46.2)		
Phone in health care facility	21	7.0 (4.1–9.9)		
No phone access	2	0.7 (0–1.6)		
**Used mobile money before enrolling for the MMHW** (N = 299)				
Yes	122	40.8 (35.2–46.4)		
No	175	58.5 (52.9–64.1)		
**Saved money before enrolling for the MMHW** (N = 294)				
Yes	200	68.0 (62.7–73.6)		
No	93	31.6 (26.3–36.9)		
**Saved money especially for health before MMHW (N = 199)**				
Yes	92	46.2 (39.3–53.2)		
No	62	31.2 (24.7–37.6)		
**Causes of not saving money before enrolling for the MMHW (N = 93)** *				
Lack of money	66	71.0 (61.7–80.2)		
No appropriate saving tool	5	5.4 (0.8–10.0)		
Other	8	8.6 (2.9–14.3)		

MGA, Malagasy Ariary; MMHW, Maternal Mobile Health Wallet. Missing values to 100%: don’t know/no answer;

*multiple answers.

Around half of women owned a mobile phone (i.e. feature phone) before the intervention. If women did not own a phone, they either had access to a phone owned by a family member, usually (40.6%; 127/300; 95% CI 35.1–46.2) or a FBHW (7.0%; 21/300; 95% CI 4.1–9.9). Two out of five (40.8%; 122/299; 95% CI 35.2–46.4) women had already used mobile money before enrolling for the MMHW. Around a third (31.6%; 93/294, 95% CI 26.3–36.9) of women had never saved any money before enrolling. The most common reason for not saving was insufficient income (71.0%; 66/93; 95% CI 61.7–80.2).

**Fig 3:**
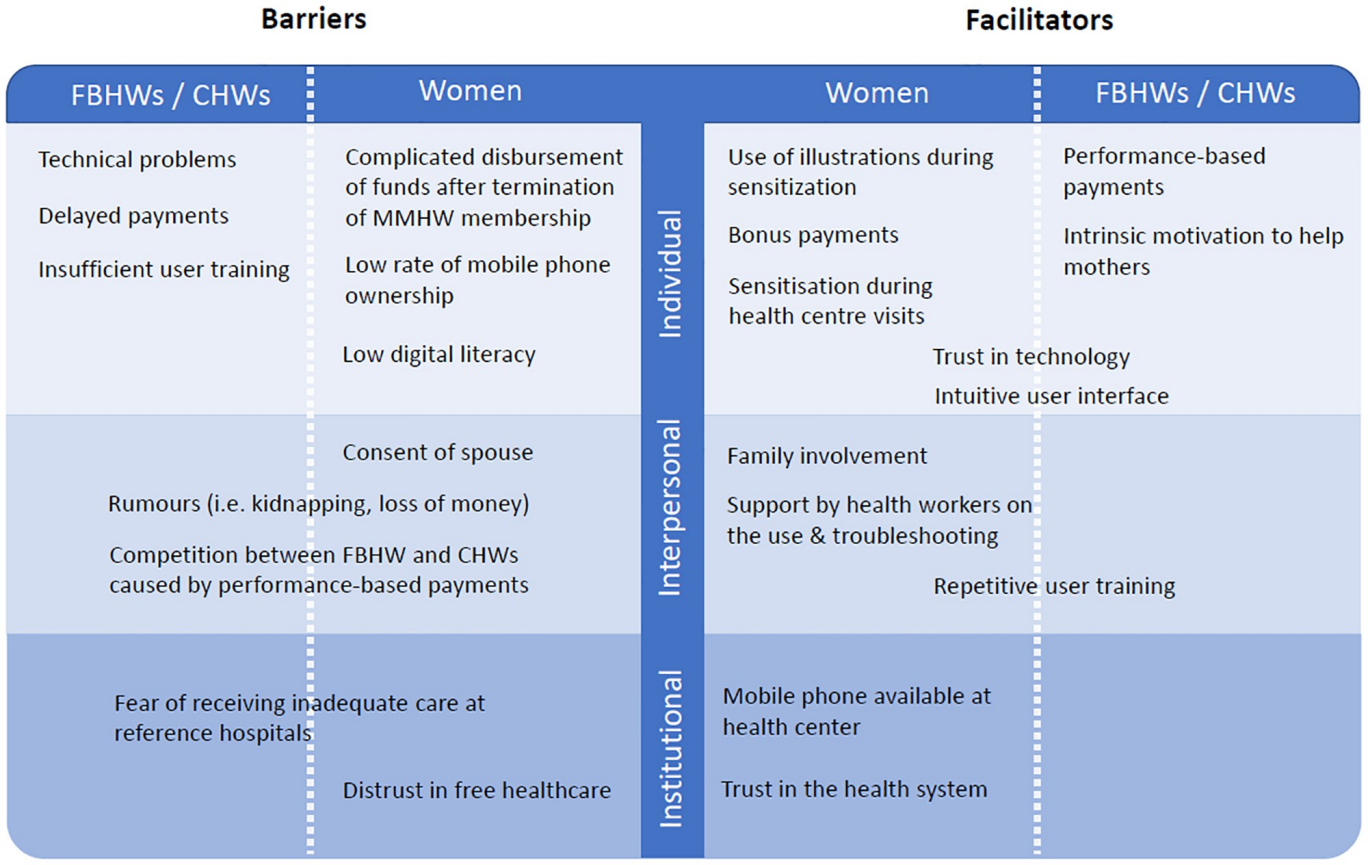
Institutional, interpersonal, and individual level factors influencing the usability and acceptance of the Mobile Maternal Health Wallet among women, facility- (FBHWs) and- community-based (CHWs) health workers.

In Fig 3, we show a summary of factors which emerged from qualitative interviews influencing the acceptance or usability of the MMHW intervention or its implementation. We show both barriers (left panel) and facilitators (right panel) related to the usability and acceptance of the MMHW during its implementation, separately for women and healthcare workers, and categorized by institutional, interpersonal, and individual level factors.

For both the usability and acceptance of the MMHW intervention, we first present descriptive statistics from the quantitative surveys, and then present results from our qualitative analyses.

### Acceptance

#### Factors influencing the acceptance of the Mobile Maternal Health Wallet among women

Three prominent themes regarding influencing factors for the MMHW’s acceptance among women emerged from qualitative interviews: i) sensitization and awareness, ii) household decision making, and iii) trust in the intervention.

### Sensitization and awareness

Target group sensitization was almost exclusively performed by FBHWs (48.2%; 145/301, 95% CI 42.5–53.8) and CHWs (43.1; 130/301, 95% CI 34.0–52.4). Sensitization almost entirely relied on verbal communication (93.0%; 292/314, 95% CI 90.2–95.8) even though visual materials in the form of flyers, posters, and brochures were available at all health facilities participating in the intervention. The most common setting for sensitization were focus group discussions (68.2%; 204/299, 95% CI 62.9–73.5) at primary health care facilities (81,9%; 245/299, 95% CI 81.9 (77.6–86.3). Sensitization was highly effective. Around three quarters of CHWs reported that women were interested in enrolling for the MMHW intervention after the first sensitization. Likewise, most women could recall all key functionalities of the MMHW intervention following sensitization. The three most common motivating factors for women to enroll for the MMHW intervention were the opportunity to save money (30.6%, 92/301, 95% CI 25.4–35.8), electronic voucher for free ultrasound 30.2%, 91/301, 95% CI 25.0–35.4), and bonus payments upon reaching a savings goal 27.9%, 84/301, 95% CI 22.8–33.0). Characteristics of sensitization activities and women’s perceptions are summarized in Table 2.

**Table 2:** Characteristics of target group sensitization activities and their perception among women.

	Variable	n	% (95%-CI)
**Women**			
	**First introduction to MMHW by** (N = 301)		
	FBHW	145	48.2 (42.5–53.8)
	CHW	48	15.9 (11.8–20.1)
	Dedicated CHW (employed by implementer)	82	27.2 (22.2–32.3)
	Friend	12	4.0 (1.8–6.2)
	Pregnant women	7	2.3 (0.6–4.0)
	Have seen commercial	1	0.3 (0.0–1.0)
	Other	4	1.3 (0.0–2.6)
	**Sensitization setting** (N = 299)		
	Group	204	68.2 (62.9–73.5)
	One on one session	88	29.4 (24.3–34.6)
	With partner	1	0.3 (0.0–1.0)
	Other	6	2.0 (0.4–3.6)
	**Location of sensitization** (N = 299)		
	Primary health care facility	245	81.9 (77.6–86.3)
	Home	35	11.7 (8.1–15.3)
	Public place	13	4.3 (2.0–6.7)
	Somewhere else	3	1.0 (0.0–2.1)
	**Found place of sensitization appropriate** (N = 300)		
	Yes	272	90.7 (87.4–94.0)
	No	28	9.3 (6.0–12.6)
	Material used for sensitization* (N = 314)		
	Verbal	292	93.0 (90.2–95.8)
	Poster	38	12.1 (8.5–15.7)
	Phone (live demo)	35	11.1 (7.7–14.6)
	Flyer	29	9.2 (6.0–12.4)
	Explanatory comics	16	5.1 (2.7–7.5)
	None	2	0.6 (0.0–1.5)
	**Material perceived as (very) helpful to understand MMHW**		
	Oral (N = 292)	262	89.7 (86.2–93.2)
	Poster (N = 36)	32	88.9 (78.6–99.2)
	Phone (N = 35)	31	88.6 (78.0–99.1)
	Flyer (N = 29)	26	89.7 (78.6–100)
	Explaining pictures (’Boîte à image’) (N = 16)	16	100.0
	**Received practical training on MMHW use** (N = 300)		
	Yes	107	35.6 (30.2–41.1)
	No	193	64.4 (58.9–69.8)
	**Training provided by** (N = 107)		
	Dedicated CHW (employed by implementer)	81	75.7 (67.6–83.8)
	FBHW	20	18.7 (11.3–26.1)
	CHW	4	3.7 (0.1–7.3)
	Other	2	1.8 (0.0–4.4)
	Knowledge of MMHW core functionalities* (N = 314)		
	Electronic vouchers for free ANC drugs	270	86.0 (82.1–89.8)
	Electronic vouchers for free ultrasound	274	87.3 (83.6–90.9)
	Electronic vouchers for free ambulance	196	62.4 (57.1–67.8)
	Save money	268	85.4 (81.4–89.3)
	Bonus payment upon reaching savings goal	275	87.6 (83.9–91.2)
	None	6	1.9 (0.4–3.4)
	Partner consent required prior to using MMHW* (N = 126)		
	Yes*	29	23.0 (15.7–30.4)
	For enrolling	7	5.6 (1.5–9.6)
	For saving	28	22.2 (15.0–29.5)
	For spending	11	8.7 (3.8–13.7)
	No need to ask partner	97	77.0 (69.6–84.3)
	**Obtaining partner consent perceived as barrier to using MMHW** (N = 29)		
	Yes	6	20.7 (5.9–35.4)
	No	23	79.3 (64.6–94.1)

MMHW, Maternal Mobile Health Wallet; FBHW, facility-based health worker; CHW, community health worker; ANC, antenatal care visit. Missing values to 100%: don’t know/no answer;

*multiple answers.

Overall, health workers stated that they could explain the MMHW intervention to women with ease. Describing the functionality of the savings component of the MMHW was perceived as the most difficult and time-consuming aspect of sensitization.

*“For me*, *it’s not difficult to explain this to women*, *but we have to repeat several times what the MMHW is before they understand*.*”* Interviewer: *“What to repeat*?*”* FBHW: *“Above all*, *explain about the account; about depositing money on the phone*. *(*…*) some people think that the money will come back to us*, *so we must explain to them that the money is not for us but for them*.*”*

(FBHW, IDI_9)

Likewise, this was reflected in qualitative interviews with women:

*“It was not really clear after the first explanation*. *Only when I heard the explanation a second or third time*, *I understood it better*.*”*

(Woman, IDI_1)

To improve sensitization, women and health workers suggested complementing verbal sensitization with flyers, posters, and TV/radio advertisements as “*explanations aren’t enough*” (Women, IDI_10). Although flyers, posters, and brochures were supplied to participating health facilities in sufficient number, these materials did not reach health workers involved in sensitization activities, indicating a need for improved follow-up.

### Household decision making

Around a quarter of women who used the MMHW reported that they needed to ask their partner for permission before they could deposit or withdraw money or access their MMHW account. Of those, 20.7% (6/29; CI 5.9–35.4) perceived the need to obtain their partner’s approval as a major barrier to acceptance. Qualitative IDIs confirmed that family involvement when deciding to enroll for the MMHW was common. In some cases, decisions concerning the enrollment to or use of the MMHW were made solely by the husband or parents. Women reported that they depend economically on their husbands, who manage the household budget and phone. Women, FBHWs and CHWs alike reasoned that husbands should, therefore, be also involved in the sensitization and training process to increase overall acceptance of the MMHW intervention. Their involvement was also deemed beneficial as husbands could use the MMHW to pay for treatment in case of an emergency when their partner becomes incapacitated.

*“The obstacle is that while you are talking to women*, *they are convinced*. *After talking to their husbands*, *they didn’t come back*. *The solution is to talk with the couple together*.*”*

(CHW, FGD_5)

Interestingly, the MMHW enabled women to be more economically independent of their partners. In cases where partners were reluctant to use the MMHW intervention, women described that the MMHW helped them to have a separate, often hidden, savings account. Women perceived the power over how and when to save money for health care as a major motivation to accept the MMHW intervention.

“*Especially for women*, *they aren’t always supposed to depend on men*. *It*’*s better to always have something for yourself*.”

(Woman, IDI_10)

### Trust in the intervention

Overall, 95.7% (287/300, 95% CI 93.4–98.0) of women and 78.9% of health workers (60/76, 95% CI 69.8–88.1) (fully) trusted the MMHW intervention. Qualitative IDIs revealed the main drivers of trust derived from trust in the public health care system and in technology itself. In turn, distrust in public health facilities or technology diminished acceptance of the MMHW intervention. Women, FBHWs, and CHWs alike reported that trust in the MMHW was undermined by rumors about the baby being “*kidnapped*”, losing money, electronic vouchers not being free of charge or fear of not being attended by the hospital staff (“*no one is looking after me*”), not receiving adequate medication, or receiving unnecessary surgical treatments.

“*We have remarked that there is some money that [the women] paid in addition apart from iron and albendazole being free*, *and on the other hand for the ultrasound*, *they started to pay also*, *the midwives in the facility ask them to pay*, *and the rumor goes that the MMHW is not for pregnant in need but for rich pregnant*.”

(CHW E, FGD_4)

One FBHW (FBHW, FGD_4) reported that the *“poorest of the poor”* are *“often hesitant because they are skeptical when something is completely free*, *especially when it includes technology*.*”*

#### Factors influencing the acceptance of the Mobile Maternal Health Wallet among facility- and community-based health workers

The most common motivating factor to participate in the MMHW intervention among FBHWs (57.9%; 44/76, 95% CI 46.8–69.0) and CHWs (84.6%; 44/52, 95% CI 74.8–94.4) was to enable women to save towards delivery, thus encouraging facility-based deliveries. Only 9.2% (7/76, 95% CI 2.7–15.7) of FBHWs and 1.9% (1/52, 95% CI 0–5.7) of CHWs perceived performance-based payments as motivating. On the contrary, respondents identified the delay of these payments among the most important factors that negatively impacted the acceptance of the MMHW among health workers as they had the feeling to “deliver for nothing” (HCP, IDI_10). In addition, health workers perceived performance-based payments as opaque and as causing unproductive competition between health workers resulting in a threat to their own income.

Experiential and motivational characteristics of the MMHW intervention by stakeholder are summarized in Table 3.

**Table 3:** Experiential and motivational characteristics to use the MMHW among women, facility- and community-based health workers.

	Variable	n	% (95% CI)
**Women**			
	Overall (very) satisfied (N = 295)	265	89.8 (86.4–93.3)
	**Would recommend MMHW** (N = 300)		
	Yes	288	96.0 (93.9–98.2)
	No	1	0.3 (0.0–1.0)
	(Fully) trust MMHW (N = 300)	287	95.7 (93.4–98.0)
	(Very) motivated to use MMHW (N = 296)	279	94.3 (91.6–96.9)
	**Main motivation** (N = 301)		
	Opportunity to save money	92	30.6 (25.4–35.8)
	Electronic voucher for free ultrasound	91	30.2 (25.0–35.4)
	Bonus payments upon reaching savings goal	84	27.9 (22.8–33.0)
	Electronic voucher for free ambulance service	11	3.7 (1.5–5.8)
	Electronic voucher for free ANC drugs	8	2.7 (0.8–4.5)
	Other	11	3.7 (1.5–5.8)
	Would save money (very) likely into MMHW even without bonus payments (N = 297)	234	78.8 (74.1–83.4)
**Facility-based health workers**			
	Overall (very) satisfied (N = 76)	59	77.6 (68.3–87.0)
	(Fully) trust MMHW (N = 76)	60	78.9 (69.8–88.1)
	(Very) motivated to use MMHW (N = 76)	49	64.5 (53.7–75.2)
	**Would recommend MMHW** (N = 76)		
	Yes	68	89.5 (82.6–96.4)
	No	8	10.5 (3.6–17.4)
	**Main motivation** (N = 76)		
	Enabling women to save money for delivery	44	57.9 (46.8–69.0)
	General interest in intervention	9	11.8 (4.6–19.1)
	Performance-based payments	7	9.2 (2.7–15.7)
	Improve quality of care	3	3.9 (0–8.3)
	Others	13	17.1 (8.6–25.6)
**Community-based health workers**			
	(Very) motivated to use MMHW (N = 52)	49	94.2 (87.9–100)
	**Main motivation** (N = 52)		
	Enabling women to save money for delivery	44	84.6 (74.8–94.4)
	General interest in intervention	3	5.8 (0–12.1)
	Performance-based payments	1	1.9 (0–5.7)
	Want to learn something new	1	1.9 (0–5.7)
	Others	3	5.8 (0–12.1)

MMHW, Mobile Maternal Health Wallet; ANC, antenatal care visit. Missing values to 100%: don’t know/no answer, not very much, not at all;

*multiple answers.

### Usability

#### Factors influencing the usability of the Mobile Maternal Health Wallet among women

Table 4 summarizes factors influencing the usability of the MMHW by user group. Three main themes emerged in qualitative interviews, which affected the usability of the MMHW among women: i) mobile phone access, ii) relationship with health workers, and iii) trust in the intervention.

**Table 4:** Factors influencing the usability of the Mobile Maternal Health Wallet by user group.

	Variable	n	% (95% CI)
**Women**			
	**Needed help to enroll for MMHW** (N = 300)		
	Yes	268	89.3 (85.8–92.8)
	No	22	7.3 (4.4–10.2)
	Never accessed the MMHW (N = 294)	168	57.1 (51.5–62.8)
	Accessed the MMHW at least once (N = 294)	126	42.9 (37.2–48.5)
	If ever accessed MMHW account, then* (N = 126)		
	Without help	72	57.1 (48.5–65.8)
	With help	82	65.1 (56.8–73.4)
	**If help needed** * (N = 82)		
	Helped by FBHW	60	73.2 (63.6–82.8)
	Helped by dedicated CHW (employed by implementer)	33	40.2 (29.6–50.9)
	Helped by husband	20	24.4 (15.1–33.7)
	Helped by CHW	4	4.9 (0.2–9.5)
	Helped by friend	1	1.2 (0.0–3.6)
	Helped by hotline	0	0.0
	**Know how to save with MMHW** (N = 299)		
	Yes	234	78.3 (73.6–82.9)
	No	59	19.7 (15.2–24.2)
	**Ever saved money with MMHW** (N = 298)		
	Yes	193	64.8 (59.3–70.2)
	No	102	34.2 (28.8–39.6)
	Used phone to save money* (N = 193)		
	From FBHW	91	47.2 (40.1–54.2)
	Own phone	60	31.1 (24.6–37.6)
	From dedicated CHW (employed by implementer)	36	18.7 (13.2–24.1)
	From Husband	14	7.3 (3.6–10.9)
	From Household	13	6.7 (3.2–10.3)
	Find saving-process (very) easy (N = 187)	183	97.9 (95.8–99.9)
	**Need help for saving process** (N = 193)		
	Yes	177	91.7 (87.8–95.6)
	No	16	8.2 (4.4–12.2)
	For saving process helped by* (N = 177)		
	FBHW	149	84.2 (78.8–90.0)
	Dedicated CHW (employed by implementer)	52	29.4 (22.7–36.1)
	Husband	15	8.5 (4.4–12.6)
	CHW	6	3.4 (0.7–6.1)
	Family	4	2.3 (0.1–4.4)
	**Find paying with MMHW (much) easier than with cash** (N = 241)		
	Yes	225	93.4 (90.2–96.5)
	No	16	6.6 (3.5–9.8)
	**In case of problems or questions concerning MMHW women consulted or would consult** (N = 300)		
	Dedicated CHW (employed by implementer)	118	39.3 (33.8–44.9)
	FBHW	77	25.7 (20.7–30.6)
	CHW	36	12.0 (8.3–15.7)
	Hotline	19	6.3 (3.6–9.1)
	Friends	10	3.3 (1.3–5.3)
	Other	8	2.7 (0.8–4.5)
**FBHW**			
	**Usability of web-based interface** (N = 28)		
	(Very) easy to use	23	82.1 (68.0–96.3)
	(Very) easy to orient	21	75.0 (59.0–91.0)
	In case of problems or questions concerning FBHW consulted or would consult* (N = 76)		
	Hotline	28	36.8 (26.0–47.7)
	Dedicated CHW (employed by implementer)	51	67.1 (56.5–77.7)
	Other	2	2.6 (0.0–6.2)
	**Additional workload perceived as (very) high**		
	for claim validation process (N = 24)	8	33.3 (14.5–52.2)
	for supporting women during savings process (N = 26)	5	19.2 (4.1–34.4)
	for supporting women during payment process (N = 27)	8	29.6 (12.4–46.9)
	Reasons to dislike the MMHW* (N = 76)		
	Additional workload	19	25 (15.8–36.3)
	Technical problems	15	19.7 (11.5–30.5)
	Late reimbursement and performance-based payments**	14	18.4 (9.7–27.1)
	Opaque bonus system	2	2.6 (3–9.2)
	Electronic vouchers (ultrasound)	2	2.6 (3–9.2)
	Free ambulance service	1	1.3 (0–3.9)
	Other	21	27.6 (17.6–37.7)
	**Ever faced a problem during payment process** (N = 24)		
	Yes	12	50.0 (30.0–70.0)
	No	12	50.0 (30.0–70.0)
	**MMHW payments perceived easier than cash** (N = 24)		
	Yes	10	41.7 (21.9–61.4)
	No	13	54.2 (34.2–74.1)

MMHW, Maternal Mobile Health Wallet; FBHW, facility-based health worker; CHW, community health worker. Missing values to 100%: don’t know/no answer, not very much, not at all;

*multiple answers.

### Mobile phone access

Only a third of women (31.1%; 60/193; 95% CI 24.6–37.6) used their own phone to save money via the MMHW, whereas most women only had access to a SIM card, which they inserted into a facility-based health worker’s (47.2% (91/193, 95% CI 40.1–54.2) or community-based health worker’s (18.7%; 36/193, 95% CI 13.2–24.1) phone to access and use the MMHW. Women perceived this as a major hindrance. Moreover, in qualitative interviews, several health workers reported that keeping the SIM card safe was difficult for women and that cards sometimes got lost causing women to lose their savings.

### Relationship with health workers

Almost all women needed help by a health worker to enroll for the MMHW (89.3%, 268/301, 95% CI 85.8–92.8). Less than half of women accessed their MMHW account at least once (42.9%, 126/294, 95% CI 37.2–48.5). However, two thirds of women used the MMHW to save money (64.8%, 193/298, 95% CI 59.3–70.2). Surprisingly, almost all women perceived the savings process as (very) easy (97.9%, 183/187, 95% CI 95.8–99.9). Likewise, most women reported that paying or redeeming an electronic voucher for maternal health services at a health facility was easier than paying by cash (93.4%; 225/241; 95% CI 90.2–96.5). Thus, many more women had used the MMHW than had ever accessed their MMHW account themselves. This indicates that most women relied on support by a health worker to either save or spend money using the MMHW. Among health workers, FBHWs were most likely to support women accessing their MMHW account (73.2%, 60/82, 95% CI 63.6–82.8) and to render support during the savings process (84.2%; 149/177; 95% CI 78.8–90.0).

Qualitative interviews revealed that women who did not have prior access to a mobile phone perceived the MMHW intervention as complex; they perceived cash as easier to use and more palpable. Several women described how over the course of the intervention they familiarized with the MMHW requiring less support from health workers.

“*I was not familiar with mobile phones*, *I always needed assistance*. *That was the small obstacle for me*. *But now*, *my husband and I know how to use it*. *Finally*, *it’s not that complicated*.”

*(Women, IDI_1)*

Only few women were aware of a toll-free support number, which was available to all users; none of them had ever called. Consequently, several women reported that if they made bad experiences with a health worker while using the MMHW, they would stop using the MMHW altogether as they wouldn’t know where else to seek support.

According to one FBHW, the MMHW intervention was preventing health workers from engaging in corruption. However, CHWs and women reported that in some cases, women were asked to pay additional services fees to FBHWs in cash. This resulted in some women feeling deceived by health workers. These women also questioned the overall usefulness of the MMHW.

### Trust in the intervention

A recurring challenge identified from qualitative interviews influencing usability of the MMHW among health workers was trust that savings to the MMHW were secure. Thus, a slow and complicated disbursement process of funds remaining in the wallet after delivery negatively affected their perception and, ultimately, use of the MMHW. According to CHWs, there were rumors that the money would not be disbursed at all, reducing overall trust in the MMHW and impeding CHWs to motivate women to use the MMHW. Overall, women wished to continue using the MMHW after childbirth also for their next pregnancy.

#### Factors influencing the usability of the Mobile Maternal Health Wallet among facility- and community-based health workers

Overall, the usability of the web based MMHW application for payments was ranked high; FBHWs (82.1%; 23/28; 95% CI 68.0–96.3) found the tablet application (very) easy to use for payments. Different from women using the MMHW, less than half of health workers (41.7%, 10/24; 95% CI 21.9–61.4) found paying via the MMHW easier than paying in cash. The main themes impacting the usability of the MMHW for health workers were i) technical problems and ii) lack of training.

### Technical problems

Half of health workers (50.0%; 12/24; 95% CI 30.0–70.0) encountered problems at least once during the payment process, which were mostly related to poor internet connection or insufficient internet credit. CHWs and women reported that technical difficulties during the payment process (internet connectivity or operational difficulties) required women to pay in cash or the treatment being delayed until payment was successful. One FBHW recommended to refrain from using the MMHW until technical and infrastructural conditions were to be improved:

*“In the health center of [*…*]*, *the connection is not working well*. *They are waiting too long*. *People do come there not for a whole day*! *And the time that people take to come there is a very precious time*. *They stayed there today but not next time*, *and the problem is that they speak to their friend about it and it will affect the MMHW program in general*.*”*

*(CHW B, FGD_4)*

### Training

Most health workers perceived the training on the use of the MMHW, which was conducted by dedicated CHWs as (very) beneficial (78.6%; 22/28, 95% CI 63.4–93.8). Nevertheless, several FBHWs expressed their wish for further and ongoing training on the MMHW in qualitative interviews. Mistakes in the use of the MMHW led to financial loss to health facilities, which, one FBHW, perceived as a “*punishment*”. Overall, health workers perceived the usability of the MMHW to increase over time.

*“I have to admit*, *it was not really clear at the beginning*. *But with time*, *as we practiced*, *I could understand how the MMHW works*. *It began with a meeting with all the people who will sensitize about the MMHW*, *and I didn’t get what the phone had to do with it*. *Only when I saw it at work that it has become clear*.”

*(IDI, 3_HCP)*

## Discussion

We identified facilitators for and barriers to the usability and acceptance of the MMHW among women and FBHWs and CHWs in Madagascar. The main factors driving acceptance and use of the MMHW among women were the opportunity to save money for health, electronic vouchers for free ultrasound exams, and bonus payments upon reaching a savings goal. Health workers were motivated by the opportunity to enable women to save towards delivery, thus encouraging facility-based deliveries. A good relationship between women and health workers was deemed essential by all groups to promote trust in the intervention. Among women, we found sociodemographic (i.e., phone ownership), educational (i.e., low digital literacy), and cultural factors (i.e. gender inequity in household decision making) to be critical barriers to acceptance and use. Among health workers, technical challenges (e.g., internet connectivity), slow payout processes, and increase in workload were common barriers.

Perhaps our most important finding was the essential role public-sector health workers had for the success of the intervention. Health workers were of great importance for enrolment, use, and equity of the MMHW. Women usually joined the MMHW program, if a health worker recommended joining. In addition, health workers were instrumental to guide and assist women to enroll and use the MMHW who could not understand or use the MMHW independently. In addition, women who did not have access to a personal phone, relied on the phone of a health worker to access their MMHW account. To our surprise, many women reported to be using the MMHW but either did not own a phone or had never even accessed the MMHW account themselves, further underscoring the fundamental role of health workers. Health workers also promoted the equity of the intervention, which is illustrated by the fact that the socio-demographic characteristics of women using the MMHW were very similar to those of pregnant women seeking care at public health facilities in Antananarivo [35]. Therefore, the design of the MMHW was inclusive for women from all socioeconomic backgrounds, including women who lacked basic education or the means to afford a mobile phone; the intervention did not create additional health inequities.

This health worker dependency also has its downsides: If a health worker is demotivated, relocates, or quits, this might create a bottleneck affecting a large group of women who will be unable to interact with the program. In addition, the dependence on a health worker may preclude women from gaining financial independence. Surprisingly, dependency did not seem to reduce trust in the intervention. Instead, most women experienced the contact with a health worker as beneficial and considered it an essential element of trust in the intervention. Studies on digital health interventions for maternal care from other countries in SSA showed a similar need for a close patient-health worker relationship to ensure women’s trust in the intervention [36].

There were mixed results on health workers’ perspectives on the usability and acceptance of the MMHW. Overall, health workers were convinced about the usefulness and benefit of the intervention, particularly the intervention’s impact on increasing facility-based deliveries. Most health workers readily explained the intervention to potential users and supported them during use. However, health workers found that they did not have sufficient time, as these tasks were additive to their already high workload. In addition, to fulfill their supporting role in this intervention, health workers wished to handle the MMHW with confidence, which they found challenging in the face of limited training opportunities. Complex interventions and interventions adding to the workload of health workers in resource-restricted settings are likely to fail [37–39]. Health workers recommended strengthening their role in the intervention through the expansion of practical training, better training materials, as well as higher and more timely payouts of performance-based payments. To grow the impact of the intervention itself, they recommended expanding the intervention to users other than pregnant women and exempting women with low socio-economic status from any user fees.

Financial incentives including performance-based payments, bonus payments, and electronic vouchers were not essential and might have even been counterproductive. Confirming our prior findings on the use of incentives to promote acceptance of the MMHW, most women and health workers perceived performance-based payments and bonus payments as a main motivation to enroll [35]. Interestingly, the majority of women would have used the MMHW even if there had been no bonus when reaching a savings goal, indicating a high level of usefulness of the intervention. On the contrary, financial incentives might have deterred women from using the MMHW. Women were inclined to deposit funds on the wallet before using electronic vouchers, which indicates that receiving free services without any contribution is uncommon in this context. This is reflected by findings from other countries in SSA where free maternal health services led to a poor perception of service quality and distrust of the health care system [40–42]. For health workers, performance-based payments were rarely reported as the main motivator to encourage women to register to the MMHW. Health workers reported to be motivated by ensuring women’s access to benefits of the MMHW. Surprisingly, this was similar for paid FBHW and volunteer CHWs.

An important finding from our study was that gender inequity in household decision making was both a major obstacle and a facilitator for enrollment and use. Women reported the need to include husbands in the decision on to enroll and to use the MMHW; if husbands were not convinced of the intervention, women could not enroll. However, once husbands were convinced of the benefit of the intervention, they, in turn, facilitated the intervention. This insight mirrors findings from other countries in SSA, which showed the importance of male involvement in facilitating access to maternal care and the influential role men play in decision making [43, 44]. Therefore, we suggest that sensitization activities should be particularly designed to enhance the accessibility of the program.

All groups experienced skepticism in the form of rumors about the motives of the intervention and the risk of fraud to be highly endangering the trust in and success of the intervention. Public health programs have faced similar challenges elsewhere [45]. Our results suggest that solutions to tackle this issue should include increased information and understanding of the intervention among all stakeholders, establishing continuous social listening strategies for early awareness of misconduct or harmful actions (i.e., free line for CHWs to report fraud), constant fact-checking, and targeted actions.

Our study had limitations. First, only women who were known to a CHW were included. Women without contact with a CHW but using the MMHW might have different perceptions and needs. This sampling strategy precluded us from determining whether the intervention reached women without former access to the health sector. However, we deliberately chose this sampling strategy as there was no alternative to retrieve participants otherwise. Second, although data collectors and interviewers were supposed to appear neutral and independent from the implementation team, some responses were unexpectedly positive. Therefore, we cannot rule out response biases, including acquiescence bias (i.e., predominantly saying yes to questions where the participant is not sure what to answer) and courtesy bias (i.e., participant is reluctant to state unhappiness). These biases might have motivated women not to lose benefits in the program when stating an undesired response.

In conclusion, we find that women and health workers were generally in favor of the MMHW intervention; respondents described a high degree of acceptance, ease of use, engagement, trust, and satisfaction with the MMHW. For the success of this intervention a close women-health worker relationship was essential. Therefore, health worker participation and ownership are paramount, and partners must be involved at all stages of sensitization and training. More research is needed to determine the impact of the MMHW intervention on access to health care, financial risk protection, and health outcomes, as well as its cost-effectiveness.

## Supporting information

S1 File (DOCX)

## Abbreviations

ANC: Antenatal care visit
CHW: Community health worker
FBHW: Facility-based health worker
FGD: Focus Group Discussion
GDP: Gross domestic product
HCP: Health care provider
HDI: Human Development Index
IDI: In-depth interviews
MMHW: Mobile Maternal Health Wallet
OOP: Out-of-pocket payments
SSA: Sub-Saharan African
